# Efficacy and Safety of Early Mobilization and Factors Associated with Rehabilitation After Stroke—Review

**DOI:** 10.3390/jcm14051585

**Published:** 2025-02-26

**Authors:** Karolina Filipska-Blejder, Krystyna Jaracz, Robert Ślusarz

**Affiliations:** 1 Neurological and Neurosurgical Nursing Department, Faculty of Health Science, Collegium Medicum in Bydgoszcz, Nicolaus Copernicus University in Toruń, 85-821 Bydgoszcz, Poland; robert_slu_cmumk@wp.pl; 2 Department of Neurological Nursing, Faculty of Health Science, Poznań University of Medical Sciences, 60-806 Poznań, Poland; jaracz@ump.edu.pl

**Keywords:** stroke, early mobilization, stroke recovery, neurorehabilitation

## Abstract

**Background/Objectives:** Knowledge about the safety and effectiveness of early post-stroke mobilization and its correlation with various factors is necessary to select an appropriate rehabilitation program and reduce the time of convalescence. Understanding the above processes will help to effectively lower the economic burden. Thus, we conducted a review to assess the safety and effectiveness of early post-stroke rehabilitation and the impact of various factors on the course of therapy. **Methods:** The analysis included publications meeting the inclusion criteria published in the years 2015–2024 in Web of Science, Scopus, Embase, and PubMed. Finally, 12 studies were qualified for the review. The study group ranged from 37 to 2325 people. **Results:** The results of studies on early stroke mobilization indicate possible benefits, including reduced time of hospitalization and faster achievement of higher functional scores. It has been shown that the important factors correlating with the effectiveness of therapy include: rehabilitation intensity, age, functional status before the stroke, depression, social support, lesion location, lower extremity deep vein thrombosis, cognitive disorder, dysphagia, and lower limb spasticity. **Conclusions:** There is a strong need for research into post-stroke rehabilitation to speed up recovery times and reduce the economic burden on the country. Current research findings on the efficacy and safety of early rehabilitation are inconsistent. There is a strong need for international guidelines.

## 1. Introduction

According to the American Stroke Association: “A stroke occurs when a blood vessel that carries oxygen and nutrients to the brain is either blocked by a clot or bursts (or ruptures). When that happens, part of the brain cannot get the blood (and oxygen) it needs, so it and brain cells die” [1]. Stroke is a disease with a high risk of death. The World Health Organization estimates that 15 million people worldwide suffer a stroke each year, and 5 million of them die. Another five million people struggle with many permanent consequences of stroke, such as paresis, paralysis, cognitive impairment, epilepsy, and aphasia. The permanent disability of patients significantly burdens the community and family [2,3]. It is recognized that the direct clinical consequences of stroke are associated with many more or less known medical, psychosocial, and musculoskeletal problems [4]. The goal of post-stroke therapy is primarily to enhance the functional and structural reorganization of the brain. Therefore, healthcare workers and scientists are still looking for effective methods to stimulate the natural healing process, which is influenced by many factors, e.g., the area of damage and its location in the brain, the patient’s condition before the stroke, genetic factors, and comorbidities. Currently, rehabilitation is the only form of treatment that is considered an effective way to enhance the healing process both in the subacute and chronic period after vascular incidents [5,6].

Stroke remains the leading cause of death and disability worldwide. The economic costs of caring for stroke patients are high. Nearly 34% of global health expenditure is spent on stroke [7,8]. Nevertheless, implementing early rehabilitation after stroke contributes to reducing the costs of care for neurological patients. Early mobilization is defined as out-of-bed activities in the acute stroke phase [9]. In many countries, patients are qualified for rehabilitation after 24 h from the moment of stroke. In general, it is worth noting that the optimal time to start physiotherapy is still elusive and is subject to many trials and studies. Both early and long-term and intensive rehabilitation play a significant role [10]. One study conducted in Washington among 72 patients showed that the respondents in the group of therapy initiated 2–3 months after the stroke were characterized by the greatest improvement one year after the stroke. The authors of the study emphasize the need to provide patients with more intensive physical rehabilitation in the period from 60 to 90 days after the stroke [11]. In turn, the primary justification for early rehabilitation is to prevent or reduce the risk of complications (falls, infections and deep vein thromboembolism, loss of cardiovascular fitness, muscle atrophy), promoting brain recovery. Furthermore, there are a number of doubts regarding early mobilization due to, among others, its possible impact on blood pressure. These concerns apply mainly to patients with hemorrhagic stroke and patients treated with recombinant tissue-type plasminogen activator (r-tPA). Many of the previously published research results on the efficacy and safety of early mobilization after acute stroke are inconsistent [12]. The A Very Early Rehabilitation Trial (AVERT) [13] series of studies highlighted that very early rehabilitation after stroke is not always beneficial. These studies also included patients who had received rt-PA. The authors of the study pointed out that, as usual, care in a stroke unit varies depending on the location. Therefore, it would be an oversimplification to simply advise usual care. However, another multicenter study, the Early Sitting in Ischemic Stroke Patients (SEVEL) [14], showed that sitting exercises over 24 h can reduce neurological deficit both at discharge and within 3 months after stroke. Due to the large discrepancies in the results of the studies conducted, the most reasonable solution would be to introduce the required safety criteria taking into account the patient’s condition, type of stroke, treatment applied, blood pressure measurement, and others. Due to the fact that the current discussion lacks a common understanding regarding the definition of early rehabilitation, evidence regarding patient qualifications, benefits, and risks of its implementation, we decided to conduct a general review of currently published studies. The aim of this review of studies was to assess the impact of the start time of rehabilitation, its effectiveness, and the correlation of multiple factors in terms of physiotherapy on the overall improvement in the functioning of patients after stroke.

## 2. Materials and Methods

### 2.1. Search Strategy

The research procedure was as follows: (1) research planning, (2) literature and research search, (3) literature selection and choice, (4) analysis of collected data and results, and (5) discussion and conclusions. The databases Web of Science, Scopus, Embase, and PubMed were searched by two independent researchers. The following key words were used in the search process: stroke, rehabilitation, early rehabilitation, clinical perspectives. There were no restrictions on the year of publication. However, we tried to ensure that the time frame of the included studies was based on recent advances in rehabilitation techniques. The databases were reviewed between August 2024 and December 2024.

### 2.2. Inclusion and Exclusion Criteria

The inclusion criteria of the systematic review were as follows: (1) presentation of original results, (2) population: stroke patients who were qualified for rehabilitation, (3) publication of results regarding mortality, adverse events, severity of stroke, and physical function indicators, (4) reporting short and/or long-term outcome, (5) full reports and research results published, (6) publications were in English.

The exclusion criteria were: (1) publications whose text was unavailable, (2) unclear research methodology, (3) lack of appropriate outcome measures, (4) reports, abstracts, case reports, reviews.

### 2.3. Data Extraction

Based on the inclusion and exclusion criteria, two independent researchers searched the available literature. The third researcher then reviewed and revised the literature selection. Important information was extracted from each eligible study, including: population, age of study participants, tools used to assess outcomes, start-up time of early rehabilitation, details of the intervention, and published key results and conclusions. Based on the extracted data, two Tables were created. The first Table assessed the impact of the time of starting rehabilitation after stroke on the final outcome and the effectiveness and safety of the intervention. The second assessed the impact of various clinical, social, and psychological factors on the quality and effectiveness of exercise interventions.

## 3. Results

### 3.1. Overview of Studies

**Figure 1:**
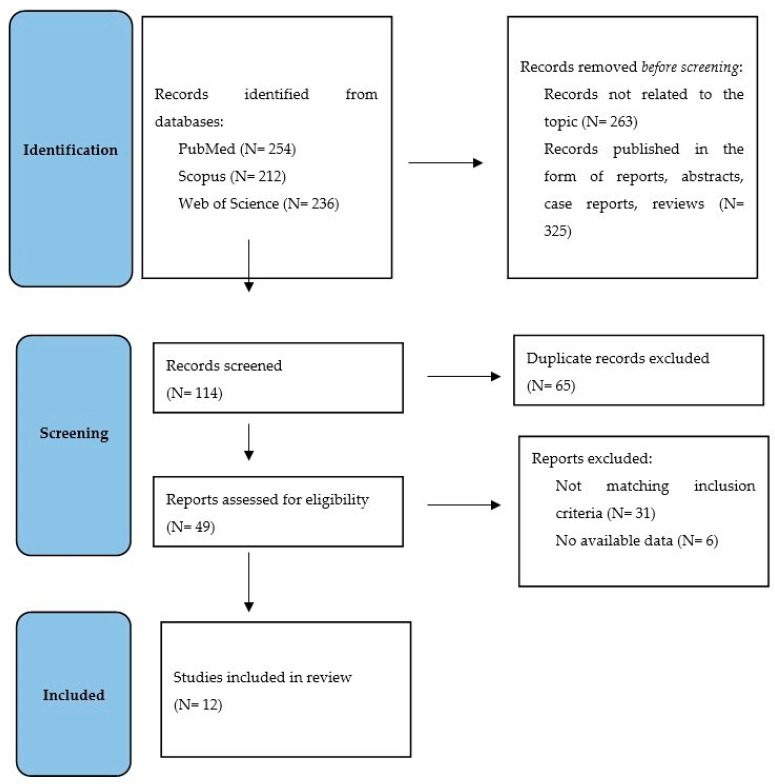
PRISMA flow diagram of the literature search strategy.

As seen in Figure 1 [15], the initial search included 702 results, of which 588 were removed because they were not related to the topic (N = 263) and publications in the form of reports, abstracts, case reports, or reviews (N = 325). Then, the records were checked for duplicates, and 65 were removed. After further comprehensive review of the articles, 49 manuscripts were excluded due to not matching inclusion criteria (N = 31) or no available data (N = 6). Finally, our review examined 12 studies that collectively examined a diverse population of stroke patients and addressed two distinct but closely related issues.

### 3.2. Characteristics of the Studies of Early Rehabilitation After Stroke

As seen in Table 1, the analyzed studies on early rehabilitation after stroke were published between 2015 and 2024. The study population was diverse and ranged from 37 [16] to 2104 [17] people. The mean age of the study participants ranged from 54 [18] to 71 [17] years. In order to assess the effectiveness and possible complications of the implemented early rehabilitation, various measurement tools were used, such as: the Functional Independence Measure (FIM) [19,20], the Postural Assessment Scale for Stroke Patients (PASS) [14,15], modified Rankin Scale (mRS) [16,21], and Barthel Index (BI) [16,21]. Additionally, measurements were taken at different time intervals, including 3, 6, and 12 months. The average time to implement early rehabilitation after stroke ranged from 24 to 48 h.

The studies included in our study assessed the effect of early rehabilitation on patients with ischemic stroke treated with thrombolysis or thrombectomy [19,21]. Yen et al. [19] showed that both patients treated with thrombolysis and mechanical thrombectomy showed improvement in the PASS scale and the motor domain of the FIM. It should be noted that only patients after intravenous thrombolysis within one month after stroke had better FIM-motor performance compared to the control group. Furthermore, in the study conducted by Wang et al. [21], the safety and efficacy of early rehabilitation in post-stroke patients using mechanical thrombectomy were assessed. It was observed that non-fatal complications after a 3-month follow-up period concerned almost 29% of the early rehabilitation group and 57% of the conventional rehabilitation group (OR 3.740, 95% CI 1.604–8.718; *p* = 0.002). The most common complications include: pulmonary infection (OR 2.701, 95% CI 1.020–7.154; *p* = 0.046), vein thrombus (OR 5.488, 95% CI 1.112–27.079; *p* = 0.037). The BI scale scores were also assessed. During the 1-year follow-up, 90% of the early rehabilitation group and 58% of the control group showed independence or moderate dependence (OR 6.308, 95% CI 2.104–18.914; *p* = 0.001). The impact of early rehabilitation safety and efficacy was also assessed in other groups of patients, including those with intracerebral hemorrhage [20], for whom bed rest is a standard procedure until now. This study showed significant improvement in the aspect of independent walking in the early rehabilitation group after two weeks and four weeks in patients with intracerebral hemorrhage. Furthermore, early mobilization patients had significant improvement in FIM-motor scores at all time points assessed (*p* = 0.004). The mean time of hospital stay in a stroke center was shorter in patients who underwent early rehabilitation (86.22 ± 41.31 vs. 119.2 ± 44.44 h; *p* = 0.004). In the studies by Poletto et al. [16] and Radford et al. [18], the efficacy and safety of early rehabilitation among stroke patients were assessed. The main results indicated similar benefits of both early and standard rehabilitation. However, a trend towards better functional results was observed more often in the early rehabilitation group. Cumming et al. [17] showed that in respondents after stroke with very early and frequent mobilization had higher scores on the Physical Senses domain in the Assessment of Quality of Life 4D at three months (coefficient = 0.013; 95% CI 0.001, 0.025; *p* = 0.035).

### 3.3. Characteristics of the Studies of Factors Associated with Rehabilitation After Stroke

Table 2 analyzes research on post-stroke rehabilitation and factors influencing it published in 2019–2024. The study population was diverse and ranged from 215 [22] to 2325 [23] people. The mean age of the study participants ranged from 54 [22] to ≥80 [24] years. In order to assess the influence of many different factors on post-stroke rehabilitation, the following measurement tools were used: the FIM scale [23], the EQ-5D-3L and BI scale [25], the Multidimensional Scale of Perceived Social Support, and Questionnaire of Exercise Adherence [24]. Additionally, motivation to recover [24] and independent walking [26] were also assessed.

Kamo et al., 2019 [23] and Wattanapan et al., 2020 [25] confirmed the results presented in Table 2. These researchers observed that patients from the intensive rehabilitation therapy group had significantly higher rates of therapy effectiveness and efficiency. Additionally, Kamo et al. [24] noticed that patients in the intensive rehabilitation group showed better improvement in cognitive functions and a higher rate of discharge home. Tang et al., 2024 [26] and Wattanapan et al., 2020 [25] showed the importance of the BI score at admission as a factor influencing the effectiveness of rehabilitation. Gnanaprakasam et al., 2024 [22] observed that patients with moderate depression were less likely to adhere to exercise recommendations than those without depression. Additionally, Tan et al., 2023 [24] discovered that social support, exercise adherence, and motivation to recover were positively correlated with stroke motivation. In the Kennedy et al., 2021 [27] study, it has been shown that the factors that delayed the return to independent walking were: severe stroke, hemorrhagic stroke, right hemisphere stroke, older age, diabetes large cortical ischemic stroke, brainstem ischemic stroke, and intracerebral hemorrhage. Furthermore, Tang et al., 2024 [26] included the following factors influencing independent walking after stroke: age, lesion location, lower extremity deep vein thrombosis, cognitive disorder, dysphagia, lower limb spasticity, Functional Ambulatory Category at admission, and National Institutes of Health Stroke Scale at admission.

## 4. Discussion

Early mobilization in patients within 24 to 72 h of admission was defined as early out-of-bed activities of daily living (ADLs) [9]. In the early period after a stroke, a window of increased plasticity is observed, which is related to the brain’s enhanced response to the injury. Therefore, rehabilitation may be more effective, especially in the first days after the injury [10,28]. Post-stroke rehabilitation is a key element of recovery and regaining normal functional and cognitive capacity. Early rehabilitation in patients with acute ischemic stroke is recommended in international clinical guidelines [29]. In practice, however, this topic still raises many doubts, concerns, and controversies among healthcare workers. There are studies that indicate that early rehabilitation does not significantly affect the efficiency of patients, but on the other hand, no negative effects of its implementation have been reported [30]. On the other hand, more and more studies are being published showing positive aspects of early rehabilitation implemented in patients after stroke, even those treated with reperfusion [19,21]. There is still a large gap regarding the safety and effectiveness of early mobilization, especially in the group of patients after hemorrhagic stroke and those treated with reperfusion. On the other side, there are studies on the impact of early rehabilitation on the rates of return to work in patients after stroke, which is an important aspect in the field of public health and health care economics [18]. In addition, research indicates that rehabilitation helps reduce symptoms such as shortness of breath, improves functional capacity, increases quality of life, and reduces the risk of depressive disorders by reducing the degree of disability [31]. Neurological rehabilitation prevents orthostatic disorders and improves motor functionality and mental state. This reduces direct and indirect costs associated with stroke and allows patients to function in social life. Moreover, scientists and healthcare workers point to the importance of implementing rehabilitation programs with the participation of a caregiver, which is associated with a high potential for improving the results and effectiveness of the therapy. Often, caregivers are actively involved in the rehabilitation process, which can have a positive impact on the sense of bond and help reduce the level of caregiver burden and facilitate the patient’s discharge from the hospital to the home environment. As a result, the time of hospitalization can be reduced, and the patient can more quickly adapt to life in a social environment and daily activities [32]. The involvement of the entire multidisciplinary team also plays an important role [28]. Currently, there is no international agreement on the introduction of early rehabilitation as a standard procedure. The American Stroke Association [12] indicates early acceptance of early rehabilitation but emphasizes the need for further research in this area and caution when qualifying patients. Post-stroke rehabilitation should be tailored to the individual needs of the patient, their clinical condition, and should include the activities of various specialists. The definition itself is also controversial. More detailed research is still required [9,10,12]. The studies we have analyzed mainly indicate the benefits of early mobilization. Furthermore, it is necessary to introduce international guidelines and required criteria for qualification and safety for patients after stroke. I believe that these criteria should apply to three main groups of patients: (1) after ischemic stroke, (2) after hemorrhagic stroke, and (3) after reperfusion therapy.

There is also another side to the effectiveness of the rehabilitation therapy. Namely, these are factors that co-correlate and influence the safety and effectiveness of rehabilitation (Table 2). The mental health of patients and their attitude towards therapy play an important role in the recovery process [22,24]. Nevertheless, social support correlates with the effectiveness of the exercises, so the importance of the family and caregiver factor should be emphasized [24]. The non-modifiable factors that influence the effectiveness of rehabilitation include: age, type of stroke, stroke location, BI scale results upon admission, and comorbidities [25,26,27]. Due to the fact that seizure activity occurs quite often, especially in people after several strokes, its occurrence in rehabilitation conditions was assessed. It was observed that epilepsy reduces the effectiveness of therapy and correlates with lower indicators of functional efficiency. Additionally, convalescence after stroke may be prolonged due to antiepileptic over-treatment [33]. Studies indicate that the involvement of medical personnel also has a significant impact on the effectiveness of rehabilitation. Mangalabarathi et al. [34] observed that rehabilitation conducted by nurses is more effective in patients after stroke. Patients achieved higher rates of functional capacity in the scope of daily activities and consciousness. Consequently, better functional and mental performance is associated with higher quality of life indicators. The important role of medical equipment and devices is also worth noting. Huang et al. [35] evaluated the use of an exoskeleton robot in gait training in early post-stroke patients. Preliminary results indicate positive effects in terms of recovery of gait speed and parameters and improvement in activities of daily living. Furthermore, Borboni et al. [36] evaluated the effect of a robot on passively assisted hand movements in patients after acute stroke. The results indicate a significant reduction in wrist swelling and pain. Moreover, it was shown that in the group of people with partial paralysis, the reduction of wrist swelling was significantly greater compared to those with complete paralysis. Finally, it is worth emphasizing that in the field of rehabilitation, the range of factors influencing its effectiveness is wide, starting from non-modifiable factors, through social support, mental health, involvement of medical personnel and/or family members, to medical equipment. Knowledge and awareness of the importance of the above-mentioned factors will help to improve the process of therapy and recovery.

## 5. Conclusions

In summary, stroke is one of the leading causes of death, physical disability, and economic burden. Implementation of an appropriate rehabilitation program in stroke patients is a key aspect in the fastest possible recovery. This review is in line with the current development trend of post-stroke rehabilitation. Knowledge of the effectiveness and safety of early mobilization and factors influencing it is essential, especially among healthcare workers involved in the recovery process of stroke patients. Many of the published research results on the efficacy and safety of early mobilization after acute stroke are inconsistent. There is a strong need for research in this area. It is necessary to implement international guidelines and required qualification and safety criteria for early rehabilitation of stroke patients.

## Figures and Tables

**Table 1:** Characteristics of the studies of early post-stroke rehabilitation.

Author, Year	Population	Age (Mean ± SD)	Measure	Start-Up Time of Early Rehabilitation	Characteristic of the Study Group	Assessment of Safety and Efficacy of Early Rehabilitation After Stroke
Yen et al., 2024 [19]	122 (N = 60 patients after intravenous thrombolysis and N = 62 mechanical thrombectomy)	--	FIM-motor, PASS; evaluated 2 weeks, 4 weeks, and 3 months post-stroke	24–72 h	The study included patients with first acute ischemic stroke treated with thrombolysis or thrombectomy. The rehabilitation in this study lasted 30 min/day, 5 days/week until discharge.	➢ Patients after intravenous thrombolysis and mechanical thrombectomy showed improved FIM-motor and PASS scores over time. ➢ Study group after intravenous thrombolysis with early mobilization had significantly improved FIM-motor performance within 1 month after stroke than the control patients with standard early rehabilitation.
Radford et al., 2024 [18]	583 (N = 324 qualified for Early Stroke Specialist Vocational Rehabilitation plus usual care and N = 259 qualified for usual care alone)	54.0 ± 11.12	self-reported at 3, 6, and 12 months return-to-work for ≥2 h per week at 12 months	within 12 weeks of stroke	Eligible participants were in work at stroke onset. Patients included in the study were hospitalized with new stroke and within 12 weeks of stroke.	➢ No evidence that Early Stroke Specialist Vocational Rehabilitation conferred any benefits over usual care in improving return-to-work rates 12 months post-stroke. ➢ The benefits of Early Stroke Specialist Vocational Rehabilitation were statistically more frequent among older participants and those with more post-stroke impairment.
Yen et al., 2020 [20]	60 (N = 30 early mobilization group, N = 30 standard early rehabilitation)	standard early rehabilitation—59.33 ± 13.13, early mobilization—58.77 ± 11.68	FIM-motor, PASS, FAC; evaluated 2 weeks, 4 weeks, and 3 months post-stroke	24–72 h	Participants ≤ 24 h after acute intracerebral hemorrhage were recruited. Early mobilization and standard group 30 min/day, 5 days/week until discharge. Inclusion criteria: a first-time primary acute intracerebral hemorrhage with unilateral hemiparesis/hemiplegia confirmed via computed tomography, complete independence in ADL before stroke, no contraindications to rehabilitation within 72 h of stroke onset.	➢ Early mobilization patients had significant improvement in the Functional Independence Measure-motor scores at all-time points assessed (*p* = 0.004). ➢ Improvements in FAC scores were also observed at 2 weeks (*p* = 0.033) and 4 weeks (*p* = 0.011) in the early mobilization group. ➢ The mean duration of hospital stay in a stroke center was shorter in patients who underwent early rehabilitation (86.22 ± 41.31 vs. 119.2 ± 44.44 h; *p* = 0.004).
Wang et al., 2022 [21]	103 (N = 52 early rehabilitation group, N = 51 conventional rehabilitation group)	early rehabilitation group 58 years (48–66.8) and conventional rehabilitation group—62 years (55–69)	mortality, mRS of 0–2, the incidence of non-fatal complications, BI scores; evaluated 3 months and 1 year post-stroke	within 48 h	Patients who met the study criteria, i.e., have undergone mechanical thrombectomy, with a history of stroke with an accompanying neurological deficit were included. The first out-of-bed exercises were performed within 48 h of stroke symptom onset. Early rehabilitation for a minimum of 5–10 min per session, with four sessions per day for ≥4 days a week until discharge.	➢ After 3 months, non-fatal complications were noted in nearly 29% of patients in the early rehabilitation group and nearly 57% in the conventional rehabilitation group (*p* = 0.002). ➢ Favorable outcomes (mRS, 0–2) were higher in the early rehabilitation group during 3-month follow-up (73.1 vs. 56.9%). The difference between these groups was also observed during 1-year follow-up (82.7 vs. 66%). ➢ The median BI after 3 months of follow-up in the early rehabilitation group was 100, while in the conventional rehabilitation group it was 87.5 (OR 0.924, 95% CI 0.873–0.979; *p* = 0.007). ➢ The median BI after 1 year of follow-up in the early rehabilitation group was 100, while in the conventional group it was 90 (OR 0.951, 95% CI 0.920–0.983; *p* = 0.003).
Poletto et al., 2015 [16]	37 (N = 18 early mobilization, N = 19 standard care)	65 years (mean)	NIHSS, mRS, activities of daily living, the modified BI; evaluated on day 14 or at discharge, 3 months	within 48 h	The study included patients with ischemic stroke confirmed by CT or MRI within 48 h of symptom onset. Early rehabilitation was performed 5 times a week, once a day, for approximately 30 min.	➢ The results in terms of modified Rankin Scale 0–2, degree of dependency and neurological deficit were similar in both groups. ➢ There was a trend towards better functional results in the early rehabilitation group. ➢ No complications related to early mobilization were observed.
Cumming et al., 2019 [17]	2104 (N = 1054 very early and more frequent mobilization, N = 1050 usual care)	intervention group—70.3 ± 13.0, usual care—70.9 ± 12.6	AQoL-4D at 12-months	within 24 h	Participants were recruited within 24 h of a confirmed stroke (first or recurrent, ischemic or hemorrhagic). The criteria for very early mobilization were considered to be: implementation of rehabilitation within 24 h of the stroke occurrence, out-of-bed activity, usual care plus 3 out-of-bed sessions per day.	➢ After 3 months, AQoL-4D scores were similar in the early rehabilitation and standard treatment groups. ➢ Patients with very early and frequent mobilization had higher scores on the Physical Senses domain at 3 months (coefficient = 0.013; 95% CI 0.001, 0.025; *p* = 0.035). ➢ AQoL-4D scores were shown to be similar in both the intervention and standard care groups after 12 months.

AQoL-4D—the Assessment of Quality of Life 4D; BI—Barthel Index; CT—computed tomography; FAC—Functional Ambulation Category; FIM—the Functional Independence Measure; MRI—Magnetic Resonance Imaging; mRS—modified Rankin scale; NIHSS—the National Institutes of Health Stroke Scale; OR—odds ratio; *p*—statistical significance; PASS—the Postural Assessment Scale for Stroke Patients; 95% CI—95% confidence interval.

**Table 2:** Characteristics of the studies of factors associated with post-stroke rehabilitation.

Author, Year	Population	Age	Measure	Characteristic of the Study Group	Assessment of Factors Associated with Post-Stroke Rehabilitation
Gnanaprakasam et al., 2024 [22]	215	54.41 ± 13.60	Patient Health Questionnaire-9, Stroke-Specific Measure of Adherence to Home-based Exercises, Fugl–Meyer Assessment-Upper Extremity	This study was conducted among 215 community-dwelling stroke survivors undergoing motor rehabilitation between February 2021 and January 2023.	➢ Patients with moderate depression were less likely to adhere to exercise recommendations than those without depression (OR: 0.69, 95% CI: 0.56, 0.85, *p* < 0.01). ➢ Type of exercises such as task-based exercises (OR: 1.80, 95% CI: 1.53, 2.13, *p* < 0.001) and movement-based (OR: 2.00, 95% CI: 1.80, 2.24, *p* < 0.001) had higher adherence odds compared to those not exercising. ➢ Severe impairment negatively affected adherence to exercise recommendations compared to mild impairment (OR: 0.71, 95% CI: 0.54, 0.94, *p* < 0.05). ➢ Severe impairment was correlated with an increased risk of minimal depression compared to mild impairment (RR: 11.09, 95% CI: 1.17, 105.04, *p* < 0.05).
Tang et al., 2024 [26]	778	59.00 (49.00–68.00)	independent walking at discharge was investigated	Participants were eligible for inclusion with a diagnosis of first-ever unilateral cerebral stroke and were unable to walk independently at admission and their Functional Ambulatory Category score was ≤3. Additionally, patients diagnosed with other underlying neurological diseases and/or disturbances of consciousness and/or unstable vital signs and the length of stay was <14 days were excluded.	➢ age (OR = 0.98, *p* = 0.018); ➢ lesion location (subcortical vs. cortical OR = 0.48, *p* = 0.009; both OR = 0.45, *p* = 0.015); ➢ lower extremity deep vein thrombosis (no vs. yes OR = 3.01, *p* = 0.002); ➢ cognitive disorder (no vs. yes OR = 2.60, *p* < 0.001); ➢ dysphagia (no vs. yes OR = 1.83, *p* = 0.029) ➢ lower limb spasticity (no vs. yes OR = 2.94, *p* < 0.001); ➢ Functional Ambulatory Category at admission (2 vs. 0 OR = 6.14, *p* < 0.001, 3 vs. 0 OR = 20.22, *p* < 0.001); ➢ NIHSS at admission (OR = 0.76, *p* < 0.001); ➢ BI at admission (OR = 1.06, *p* < 0.001)
Tan et al., 2023 [24]	350	60–69 years—21.4% of patients; 70–79 years—35.1%; ≥80 years—43.4%	Multidimensional Scale of Perceived Social Support, Questionnaire of Exercise Adherence, Tampa scale of kinesiophobia, Motivation in stroke patients for rehabilitation scale	The study included patients hospitalized in the stroke rehabilitation unit. The inclusion criteria for the participants were: age ≥ 60 years, diagnosis stroke, able to communicate normally, and conscious.	➢ Social support, exercise adherence, and motivation to recover were positively correlated with stroke motivation (r = 0.619, *p* < 0.01; r = 0.569, *p* < 0.01). ➢ Kinesiophobia was negatively correlated with motivation to recover (r = −0.677, *p* < 0.01). ➢ The time of stroke, location of the lesion influenced the patients’ motivation to recover.
Kennedy et al., 2021 [27]	2100	72.5 years	days to walking 50 m unassisted, tested from 24 h to 3 months post-stroke	Participants were eligible for inclusion with a diagnosis of first or recurrent stroke and admitted to a hospital within 24 h of stroke onset.	➢ Median number of days to walking 50 m unassisted was 6 days. ➢ Nearly 75% of patients began walking independently within 3 months. ➢ It has been shown that the factors that delayed the return to independent walking were: severe stroke, hemorrhagic stroke, right hemisphere stroke, older age, diabetes large cortical ischemic stroke, brainstem ischemic stroke, and intracerebral hemorrhage.
Wattanapan et al., 2020 [25]	780	61.9 ± 13.3	EQ-5D-3L and BI at admission and at discharge	Patients were classified into two groups: intensive or non-intensive rehabilitation. Intensive rehabilitation included patients who could tolerate rehabilitation for at least 3 h per day, 5 days per week.	➢ In the intensive group effectiveness and efficiency were significantly higher than in the non-intensive. ➢ Length of stay in hospital, quality of life, intensive rehabilitation significantly correlated positively with effectiveness of stroke rehabilitation. ➢ Onset to admission interval, BI at admission, age correlated negatively with the effectiveness of stroke rehabilitation. ➢ Younger participants with shorter onset to admission interval, lower BI at admission, and longer length of stay realized significantly enhanced effectiveness
Kamo et al., 2019 [23]	2325	76.8 ± 7.1	FIM	This study included patients with a diagnosis of stroke. The inclusion criteria for the participants were: aged more than equal to 65 years, stayed in rehabilitation hospital less than 180 days, had complete data. Patients were classified into two groups: intensive rehabilitation therapy and usual rehabilitation. The intensive rehabilitation therapy group included 862 stroke patients who performed more than 15 h of rehabilitation therapy per week in cooperation with a physiotherapist, occupational therapist, and/or speech therapist.	➢ Longer weekly rehabilitation time resulted in improved functioning. ➢ Patients from the intensive rehabilitation therapy group showed significantly higher cognitive and FIM-motor gain and discharge rate to home.

BI—Barthel Index; FIM—the Functional Independence Measure; NIHSS—National Institutes of Health Stroke Scale; OR—odds ratio; *p*—statistical significance; r—Spearman’s correlation coefficient; RR—relative risk; 95% CI—95% confidence interval.

## Data Availability

Not applicable.
